# Mortality in children with complicated severe acute malnutrition is related to intestinal and systemic inflammation: an observational cohort study^1^,^2^

**DOI:** 10.3945/ajcn.116.130518

**Published:** 2016-09-21

**Authors:** Suzanna Attia, Christian J Versloot, Wieger Voskuijl, Sara J van Vliet, Valeria Di Giovanni, Ling Zhang, Susan Richardson, Céline Bourdon, Mihai G Netea, James A Berkley, Patrick F van Rheenen, Robert HJ Bandsma

**Affiliations:** 3 Division of Gastroenterology, Hepatology, and Nutrition,; 4 Physiology and Experimental Medicine, Peter Gilgan Centre for Research and Learning,; 5 Microbiology Department, and; 6 Centre for Global Child Health, The Hospital for Sick Children, Toronto, Ontario, Canada;; 7 Department of Paediatrics and Child Health, College of Medicine, University of Malawi, Blantyre, Malawi;; 8 Global Child Health Group, Emma Children’s Hospital, Academic Medical Centre, Amsterdam, Netherlands;; 9 University of Groningen, University Medical Center Groningen, Department of Pediatric Gastroenterology, Hepatology, and Nutrition, Groningen, Netherlands;; 10 Department of Internal Medicine and Radboud Center for Infectious Diseases, Radboud University Medical Center, Nijmegen, Netherlands;; 11 Kenya Medical Research Institute-Wellcome Trust Research Programme, Kilifi Nuffield Department of Clinical Medicine, Oxford University, Oxford, United Kingdom; and; 12 Childhood Acute Illness and Nutrition Network (CHAIN)

**Keywords:** cytokines, diarrhea, inflammation, inflammatory bowel disease, severe acute malnutrition

## Abstract

**Background:** Diarrhea affects a large proportion of children with severe acute malnutrition (SAM). However, its etiology and clinical consequences remain unclear.

**Objective:** We investigated diarrhea, enteropathogens, and systemic and intestinal inflammation for their interrelation and their associations with mortality in children with SAM.

**Design:** Intestinal pathogens (*n* = 15), cytokines (*n* = 29), fecal calprotectin, and the short-chain fatty acids (SCFAs) butyrate and propionate were determined in children aged 6–59 mo (*n* = 79) hospitalized in Malawi for complicated SAM. The relation between variables, diarrhea, and death was assessed with partial least squares (PLS) path modeling.

**Results:** Fatal subjects (*n* = 14; 18%) were younger (mean ± SD age: 17 ± 11 compared with 25 ± 11 mo; *P* = 0.01) with higher prevalence of diarrhea (46% compared with 18%, *P* = 0.03). Intestinal pathogens *Shigella* (36%), *Giardia* (33%), and *Campylobacter* (30%) predominated, but their presence was not associated with death or diarrhea. Calprotectin was significantly higher in children who died [median (IQR): 1360 mg/kg feces (2443–535 mg/kg feces) compared with 698 mg/kg feces (1438–244 mg/kg feces), *P* = 0.03]. Butyrate [median (IQR): 31 ng/mL (112–22 ng/mL) compared with 2036 ng/mL (5800–149 ng/mL), *P* = 0.02] and propionate [median (IQR): 167 ng/mL (831–131 ng/mL) compared with 3174 ng/mL (5819–357 ng/mL), *P* = 0.04] were lower in those who died. Mortality was directly related to high systemic inflammation (path coefficient = 0.49), whereas diarrhea, high calprotectin, and low SCFA production related to death indirectly via their more direct association with systemic inflammation.

**Conclusions:** Diarrhea, high intestinal inflammation, low concentrations of fecal SCFAs, and high systemic inflammation are significantly related to mortality in SAM. However, these relations were not mediated by the presence of intestinal pathogens. These findings offer an important understanding of inflammatory changes in SAM, which may lead to improved therapies. This trial was registered at www.controlled-trials.com as ISRCTN13916953.

## INTRODUCTION

Severe acute malnutrition (SAM)^12^ in children results in unacceptably high mortality rates of ≤500,000 deaths/y worldwide, although undernutrition underpins ∼45% of all deaths in children under the age of 5 y (1). Despite standardized treatment and refeeding protocols, inpatient mortality reaches up to 30% in many hospitals (2–5). Current protocols rest on insufficient high-quality evidence (6), thus a more thorough understanding of the pathophysiology of malnutrition is required to improve outcomes in this devastating condition.

Diarrhea accompanies SAM in 47–67% of cases and has been associated with increased mortality (2, 3, 7). HIV enteropathy, environmental enteric dysfunction, acute infectious gastroenteritis, and secondary osmotic diarrhea with subsequent dehydration are diverse factors that likely contribute to the etiology of this diarrhea. SAM is associated with structural and immunologic changes in the small intestine, such as villous atrophy, increased permeability of the intraepithelial layer, and local inflammation including infiltration of lymphocytes (8, 9) and Th1-mediated upregulation of interferon γ (IFNγ) (8). These changes that occur in SAM may share a common pathophysiology with other T cell–mediated inflammatory conditions of the small intestine, such as inflammatory bowel disease (IBD) (10). Intestinal function and inflammation in SAM may be modulated by short-chain fatty acids (SCFAs), which are fermentation byproducts produced by the intestinal microflora and are an important energy source for intestinal epithelial cells (11–14). Malnutrition is also associated with altered systemic immune responses, such as abnormal proinflammatory priming (15, 16) decreased regulatory cytokines, and impaired immune activation (17–19).

The interplay between intestinal and systemic inflammation and their relation to mortality in SAM is not well known. We hypothesized that mortality in children with SAM is associated with diarrhea and both higher intestinal and systemic inflammation, which could be related to intestinal pathogens. In 79 children with SAM, we evaluated the relation between: *1*) diarrhea; *2*) a panel of intestinal pathogens (*n* = 15); *3*) calprotectin as a marker of intestinal inflammation; *4*) the SCFAs butyrate and propionate; *5*) markers of systemic inflammation (26 cytokines), and *6*) mortality.

## METHODS

### Study design and patient recruitment

We studied 79 children who were drawn from a previous randomized clinical trial originally designed to compare the outcomes of 3 commonly used WHO rehabilitation diets (http://www.isrctn.com/ISRCTN13916953). The 3 diets were isocaloric but varied in their composition of carbohydrate and fat ratios. Between January and July 2013, this study aimed to recruit 90 children aged 6–60 mo admitted to the malnutrition ward of Queen Elizabeth Central Hospital in Blantyre, Malawi. These children either failed a standardized appetite test with ready-to-use therapeutic food or had complicated SAM. Complicated SAM was defined as generalized edema including both feet, legs, hands, and arms and face and/or midupper arm circumference <115 mm or a weight-for-height or length <–3 *z* score and medical complications or “danger signs” as defined in the current WHO guidelines (6, 20). This includes respiratory distress, cyanosis, shock (delayed capillary refill with fast and weak pulse plus temperature gradient), impaired consciousness, hypoglycemia, convulsions, severe dehydration, profuse watery diarrhea, severe vomiting, and hypothermia. Children were excluded from the original cohort if they *1*) were HIV positive (or exposed, for children <18 mo of age) and had been readmitted to hospital for SAM within the past year, *2*) had a packed cell volume of <15%, *3*) had severe hemodynamic instability, *4*) had unknown HIV status (because of testing refusal by caregiver), or *5*) had severe neurologic symptoms. From this original cohort, we subsequently excluded children with confirmed or clinically suspected tuberculosis (*n* = 3), malaria (*n* = 7), or insufficient serum for analyses (*n* = 1). HIV-positive or exposed (for children <18 mo of age) children diagnosed by rapid antibody testing on admission were included in this study. All children admitted received care according to the WHO guidelines adapted to local Malawian use. This consisted of *1*) the start of a stabilization diet with WHO-standardized formula (named F-75 for 75 kcal/100 mL), *2*) antibiotic therapy with chloramphenicol and gentamicin, *3*) anti-helminthic therapy with albendazole, *4*) vitamin A and folic acid, and if no clinical improvement occurred in 48 h, 5) the addition of the antibiotics metronidazole or ciprofloxacin. Informed consent was obtained from the caregivers before original patient enrollment. Ethical approval was obtained from the College of Medicine Research Ethics Committee of the University of Malawi, College of Medicine in Blantyre, Malawi, and the Research Ethics Board of The Hospital for Sick Children in Toronto, Canada. Primary outcomes were death and diarrhea. Exposures included duration of stay, HIV reactivity, SAM phenotype marasmus compared with kwashiorkor, age, and anthropometric measures. Potential confounders included undiagnosed medical comorbidities, including nosocomial infections. The detailed study flowchart is presented in **Supplemental Figure 1**.

### Clinical data and biological sample collection

At admission, clinical, demographic and anthropometric data were recorded including appetite, weight, presence and degree of edema, and stool consistency and frequency. Death or discharge from hospital was also documented. Diarrhea was reported by maternal recall and defined as ≥3 loose or watery stools within 24 h. Stool and blood were obtained at admission. Stool was immediately cooled and then frozen at −80°C. Blood was centrifuged 2180 × *g* for 10 min at 4°C and collected serum was stored at −80°C until analyses.

### Stool pathogens

Fecal intestinal pathogens (*n* = 15) were assessed by polymerase chain reaction at the Hospital for Sick Children, Toronto, Canada, with the use of the Gastrointestinal Pathogen Panel (Luminex Molecular Diagnostics) according to the manufacturer’s instructions. In brief, nucleic acids were extracted from 100–150 mg of formed or 100 μL of loose stool by easyMAG extractor (bioMerieux) and underwent multiplexed polymerase chain reaction for *Salmonella* spp., *Shigella* spp., *Campylobacter jejuni/coli*, *Yersinia* *enterocolitica* (pathogenic serotype only), *Escherichia coli* 0157:H7, non-0157 shiga-like toxin-producing *E. coli*, *Clostridium difficile* toxin A/B, enterotoxigenic *E. coli*, *Vibrio cholerae*, rotavirus A, adenovirus 40/41, norovirus GI/II, *Giardia lamblia, Entamoeba histolytica,* and *Cryptosporidium parvum*.

### Fecal calprotectin and SCFAs

Fecal calprotectin, a marker of intestinal inflammation, was measured by standard enzyme-linked immunoabsorbent assay by the University Medical Center Groningen, Clinical Laboratory in the Netherlands. The SCFA concentrations of propionate and butyrate were analyzed by using gas chromatography–mass spectrometry (GC-MS); freeze-dried samples were reconstituted in 75% ethanol, homogenized, and centrifuged. Samples and standards [propionic (C3:0) and butyric (C4:0) acids] were acidified then esterized with pentafluorobenzyl bromide and di-isopropylamine before application to GC-MS wells. The GC Agilent 7890A and the MSD Agilent 5975C quadrupole mass detector with carrier gas of helium heated were used according to manufacturer’s protocol.

### Serum cytokines

Serum cytokine concentrations (*n* = 29) were determined by using a human cytokine magnetic bead assay (EMD Millipore) on the Luminex 200 platform with Xponent software (version 3.1; Luminex Corp.). **Supplemental Table 1** lists all cytokines assessed.

### Statistical analysis

R statistical software (version 3.2.1) was used for all analysis (21). As indicated, tables present either means ± SDs or medians (IQRs). Age- and sex-corrected *z* scores for anthropometric variables were calculated and are presented separately for children with marasmus and kwashiorkor. Univariate analysis was done with logistic regression or Fisher’s exact tests. Partial least squares (PLS) methods were used to examine the relation between diarrhea, calprotectin, SCFAs, selected markers of systemic inflammation (i.e., 9 cytokines), and death. Cytokine variables were log-transformed, mean-centered, and scaled. Variables that did not have enough variance to be analyzed (i.e., were undetected in most samples) were automatically identified and filtered out by using the nearZeroVar function implemented in the caret package (22). By using plsdepot (23), the variance relating to death or diarrhea was extracted and components assessed with Q_2_ values. Q_2_ values are a performance measure calculated by cross-validation that is based on the sum of squared errors. Q_2_ is equal to 1 minus the prediction error sum of squares divided by the total sum of squares of the response variable. The higher the value of Q_2_, the better the model is for prediction; negative Q_2_ values indicate that the components of the model are not predictive. Cytokines with correlation values of >0.30 to either death or diarrhea were tested for stability by using sparse PLS discriminant analysis as implemented in mixOmics (24). Feature stability was tested by using 10-fold cross-validation, i.e., the dataset was subdivided into 10 parts and the analysis serially repeated by using 9 of the 10 data subsets. Variables that were selected ≥90% of the time in the top 10 features associated with either death or diarrhea were considered most robust and stable. The cytokines that showed *1*) a *P* < 0.05 with logistic regression, *2*) a PLS discriminant analysis correlation with either death or diarrhea of >0.3, and *3*) a stability of ≥90% were selected to be included in the PLS path modeling. These cytokines in combination with diarrhea, calprotectin, SCFAs, and death were used for PLS path modeling with plspm (25). PLS path modeling is used to study a system of relation between multiple “blocks” of variables (i.e., relation between multiple data tables). Similar to principal component analysis, the high dimensional blocks of variables can be reduced to a few main components that can represent the node in the model, such as systemic inflammation (see **Supplemental Methods** for more information on the PLS path method). For this particular analysis, 62 patients were included because they had diarrhea status at admission and both blood and fecal samples. Variables were log-transformed, mean-centered, and scaled, and missing fields were imputed by using bag impute from the caret package (22). Diarrhea, calprotectin, SCFAs, and systemic inflammation were then related to mortality. Path coefficients were calculated for each interconnected node. These path coefficients indicate the strength and direction of the relation between nodes and can be conceptually interpreted as correlation coefficients.

## RESULTS

### Clinical characteristics, diarrhea, and mortality

In this cohort of 79 children with SAM, rates of diarrhea were modest at admission, and mortality was substantial with an overall rate of 23%. Differences in clinical characteristics of children who died compared with those who recovered are detailed in **Table 1**.

**TABLE 1:** Comparison of clinical characteristics at admission in children with severe acute malnutrition^1^

	All (*n* = 79)	Recovery (*n* = 65)	Death (*n* = 14)	*P*^2^
Age, mo	23.3 ± 11.7	24.7 ± 11.4	17.0 ± 11.2	0.01
Female	44/78 (56)	34/64 (53)	10/14 (71)	0.3
HIV reactive	28/79 (35)	21/65 (32)	7/14 (50)	0.2
Kwashiorkor	52/79 (66)	47/65 (72)	5/14 (36)	0.01
Weight at admission, kg				
Marasmus (*n* = 27)	5.1 ± 1.0	5.5 ± 0.9	4.4 ± 0.9	0.02
Kwashiorkor (*n* = 52)	8.2 ± 2.1	8.8 ± 1.9	7.1 ± 2.6	0.07
MUAC^3^ at admission, cm				
Marasmus (*n* = 27)	9.7 ± 1.0	10.0 ± 1.0	9.2 ± 0.9	0.07
Kwashiorkor (*n* = 52)	12.5 ± 1.6	12.6 ± 1.0	11.3 ± 2.4	0.09
Weight-for-height *z* score				
Marasmus (*n* = 27)	−4.9 ± 1.0	−4.7 ± 0.8	−5.4 ± 1.2	0.08
Kwashiorkor (*n* = 51)	−2.0 ± 1.5	−2.0 ± 1.4	−2.6 ± 1.6	0.3
Diarrhea on day of admission	17/73 (23)	11/60 (18)	6/13 (46)	0.03
Diarrhea within 72 h of admission	46/79 (58)	37/65 (57)	9/14 (64)	0.6
Anorexia	5.1 ± 6.3	4.86 ± 6.3	6.17 ± 6.3	0.5
Length of hospital stay, d		10.4 ± 4.1	9.6 ± 6.9	0.5

1 Values are means ± SDs or *n*/*N* (%). *P* values were obtained with logistic regression.

2 Significant at *P* < 0.05.

3 MUAC, midupper arm circumference.

Patients who died were younger and tended to have lower anthropometric measurements (i.e., body weight, weight-for-height *z* score, and midupper arm circumference; this analysis was stratified by edema status). Children who died were also more likely to have marasmus than kwashiorkor. Diarrhea within the first 24 h of admission was significantly associated with death. Although prevalence of diarrhea after treatment initiation (72 h after admission) more than doubled, it was not a predictor of mortality in this cohort. Parent-reported anorexia before admission did not differ between children who recovered or died. Two-thirds of patients were diagnosed with kwashiorkor, and one-third (35%) were HIV reactive or exposed (for children <18 mo of age). HIV status was not associated with death, and age, HIV status, and kwashiorkor were not significantly associated with diarrhea (data not shown).

### Enteric pathogens are highly prevalent, and intestinal markers of inflammation are altered in children with malnutrition

The presence of stool pathogens in relation to death is detailed in **Table 2**. Most malnourished children harbored known intestinal pathogens; of these, 44% had ≥2 pathogens. The bacteria *Shigella* spp., *C. jejuni/coli,* and the parasite *G. lamblia* occurred most often. Despite their high frequency and diversity, their presence was not significantly associated with systemic inflammation (data not shown) or with clinical outcomes of death (Table 2) or diarrhea (**Supplemental Table 2**).

**TABLE 2:** Stool pathogens in children admitted with severe acute malnutrition who recovered or died^1^

	All (*n* = 64)	Recovery (*n* = 53)	Death (*n* = 11)	*P*	FDR-*P*^2^
All pathogens					
≥1 pathogen	54 (84)	46 (87)	8 (73)	0.4	1
1 pathogen	26 (41)	23 (43)	3 (27)	0.5	1
2 pathogens	14 (22)	11 (21)	3 (27)	0.7	1
≥3 pathogens	14 (22)	12 (23)	2 (18)	1	1
Bacteria					
≥1 bacteria	40 (63)	34 (64)	6 (55)	0.7	1
* Shigella* spp.	23 (36)	20 (38)	3 (27)	0.7	1
* Campylobacter* spp.	19 (30)	16 (30)	3 (27)	1	1
Enterotoxigenic *Escherichia coli*	10 (16)	9 (17)	1 (9)	1	1
* Salmonella* spp.	5 (8)	4 (8)	1 (9)	1	1
Shiga-like toxin-producing *E. coli*	1 (2)	1 (2)	0 (0)	1	1
* Clostridium difficile*	1 (2)	0 (0)	1 (9)	0.2	1
Parasites					
≥1 parasite	23 (36)	20 (38)	3 (27)	0.7	1
* Giardia lamblia*	21 (33)	20 (38)	1 (9)	0.08	0.8
* Cryptosporidium parvum*	2 (3)	0 (0)	2 (18)	0.03	0.6
* Entamoeba histolytica*	1 (2)	1 (2)	0 (0)	1	1
Viruses					
≥1 virus	17 (27)	14 (26)	3 (27)	1	1
Norovirus	8 (13)	7 (13)	1 (9)	1	1
Rotavirus	5 (8)	3 (6)	2 (18)	0.2	1
Adenovirus	4 (6)	4 (8)	0 (0)	1	1

1 Values are *n* (%). *P* values were obtained with Fisher’s exact test. *Yersinia enterocolitica*, *Escherichia coli* 0157:H7, and *Vibrio cholerae* were undetected.

2 FDR-*P*, Benjamini & Hochberg, i.e., false discovery rate adjusted *P* values.

**FIGURE 1:**
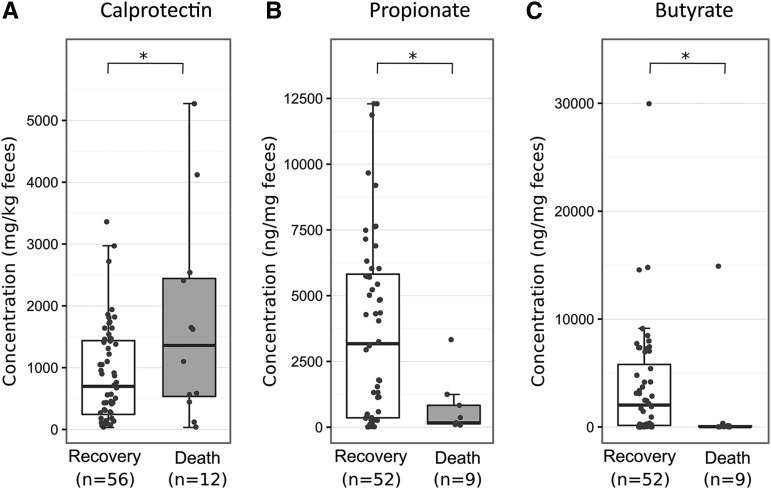
Concentrations of calprotectin (*n* = 68; A), propionate (*n* = 61; B), and butyrate (*n* = 61; C) in fecal samples from children with severe acute malnutrition who recovered or died. Boxplots summarize the median (midline) and IQRs (upper and lower boxes); overlaid dots indicate all individual data points. Medians (IQRs) for groups that recovered or died were as follows: for calprotectin, 697.5 mg/kg feces (1437.5–243.8 mg/kg feces) compared with 1360 mg/kg feces (2442.5–535 mg/kg feces, *P* = 0.03); for propionate, 3173.8 ng/mL (5819.2–357.2 ng/mL) compared with 167.2 ng/mL (831.4–130.9 ng/mL, *P* = 0.04); and for butyrate, 2035.7 ng/mL (5799.6–149.1 ng/mL) compared with 31.3 ng/mL (112.3–21.6 ng/mL), *P* = 0.02). Group differences were tested by logistic regression. *P < 0.05.

Based on an age-specific cutoff (26), fecal calprotectin concentrations were clinically elevated in the majority of patients (76%, >214 mg/kg feces) and 49% had values >800 mg/kg feces. Elevation of this intestinal inflammation marker was significantly associated with death [median (IQR): 1360 mg/kg feces (2443–535 mg/kg feces) compared with 698 mg/kg feces (1438–244 mg/kg feces), *P* = 0.03)] (**Figure 1A** and **Supplemental Table 3**). Calprotectin did not differ significantly between those with and without diarrhea (**Supplemental Table 4**); however, concentrations >800 mg/kg feces were associated with the presence of multiple intestinal pathogens (59% compared with 28%, *P* = 0.02).

Low amounts of fecal butyrate and propionate were significantly associated with death (Figure 1B and C and Supplemental Table 3), and these SCFAs were similarly decreased and highly correlated (ρ = 0.77, df = 34, *P* < 0.001). Diarrhea also tended to be associated with a decrease in SCFAs, but this was not significant when individually testing butyrate and propionate in univariate analysis (Supplemental Table 4). Also, they did not correlate with calprotectin or length of reported anorexia before admission (data not shown).

### Systemic inflammation is increased with SAM and is related to death and diarrhea

**FIGURE 2:**
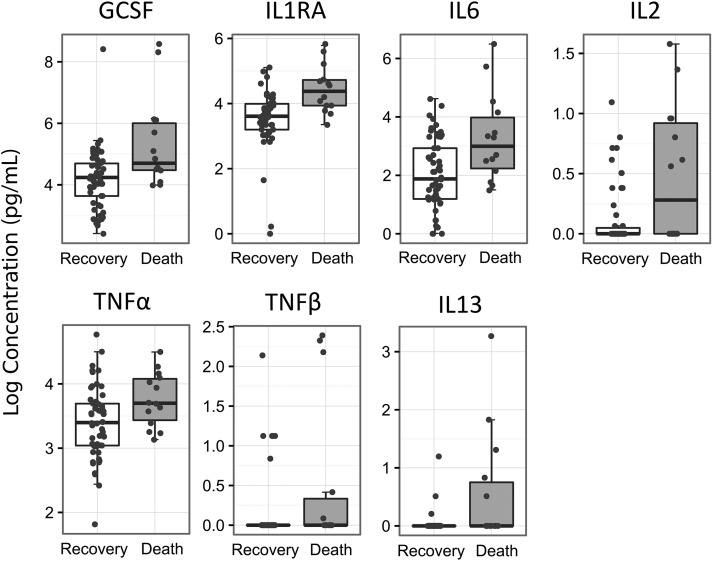
Serum cytokine concentrations in children (*n* = 68) with severe acute malnutrition who recovered (*n* = 54) or died (*n* = 14). Cytokines presented (*n* = 7) are those associated with death as obtained through partial least square–based feature selection. Boxplots summarize the medians and IQRs of natural logarithms of cytokine concentrations. Overlaid dots present all individual data points. GCSF, granulocyte-colony stimulating factor.

Several markers of systemic inflammation positively correlated with both diarrhea and death. Specific serum cytokines were found to be higher in children who died and also in children presenting diarrhea at admission (**Table 3** and **Supplemental Tables 5** and **6**). The first PLS component that captures the main variability of the cytokines with diarrhea was more modest with an R_2_ of 0.24, Q_2_ of 0.08, and prediction error rate of 0.25, whereas death had with its first component an R_2_ of 0.36, Q_2_ of 0.18, and cross-validation prediction error rate of 0.19. Most cytokines showed similar positive correlations patterns for both death and diarrhea, but their stability on cross-validation varied (Table 3). Seven cytokines were found to robustly correlate with death: granulocyte-colony stimulating factor (GCSF), IL13, IL1RA, IL2, IL6, TNF-α, and TNF-β (Table 3 and **Figure 2**). Of these, 5 also robustly correlate with diarrhea (GCSF, IL2, IL6, TNF-α, and TNF-β). These results were largely unchanged after correcting for sex and age (data not shown). Cytokines were not significantly predictive of pathogens, fecal calprotectin, or HIV reactivity and exposure as assessed by low R_2_ and Q_2_ values (data not shown).

**TABLE 3:** PLS-based feature selection of cytokines that differentiate groups of children with severe acute malnutrition who had diarrhea or died^1^

		Diarrhea		Death	
	Cytokine	Component 1 (Cor: 0.49; R_2_: 0.24; Q_2_: 0.08)^2^	Feature stability,^3^ %	Component 1 (Cor: 0.60; R_2_: 0.36; Q_2_: 0.18)^2^	Feature stability,^3^ %
1	EGF	0.31^4^	—	0.15	—
2	Eotaxin	0.14	—	−0.03	—
3	GCSF	0.64^4^	100	0.68^4^	90
4	GMCSF	0.53^4^	—	0.41^4^	—
5	IFNα2	0.59^4^	100	0.48^4^	70
6	IFNγ	0.39^4^	—	0.17	—
7	IL10	0.26	—	0.29	—
8	IL12p40	0.26	—	0.11	—
9	IL12p70	0.64^4^	100	0.55^4^	80
10	IL13	0.35^4^	—	0.5^4^	100
11	IL15	0.59^4^	—	0.58^4^	70
12	IL17A	0.08	—	0.19	—
13	IL1RA	0.65^4^	50	0.75^4^	100
14	IL1α	0.16	—	0.12	—
15	IL2	0.74^4^	100	0.74^4^	100
16	IL5	0.24	—	0.17	—
17	IL6	0.56^4^	100	0.61^4^	100
18	IL7	0.26	—	0.36^4^	—
19	IL8	0.3^4^	—	0.4^4^	—
20	IP10	0.28	—	0.14	—
21	MCP1	0.34^4^	—	0.33^4^	—
22	MIP1α	0.21	—	0.02	—
23	MIP1β	0.07	—	0.03	—
24	TNF-α	0.37^4^	100	0.33^4^	100
25	TNF-β	0.56^4^	100	0.55^4^	100
26	VEGF	0.24	—	0.23	—

1 IL3, IL4, and IL1β were not analyzed because they were undetected in most samples. EGF, epidermal growth factor; GCSF, granulocyte-colony stimulating factor; GMCSF, granulocyte-macrophage colony stimulating factor; IFN, interferon; IP, induced protein; MCP, monocyte chemoattractant protein; MIP, macrophage inflammatory protein; PLS, partial least square; VEGF, vascular endothelial growth factor.

2 Cor indicates the correlation strength between the PLS-component 1 and either diarrhea or death. R_2_ indicates the variance explained by component 1. Q_2_ indicates the predictive quality of component 1; Q_2_ is equal to 1 minus the prediction error sum of squares divided by the total sum of squares of the response variable; negative Q_2_ values indicate that the component is not predictive.

3 Feature stability indicates the percentage of times that a cytokine was selected as a top-10 feature by using sparse PLS with 10-fold cross-validation.

4 Correlation >0.3 with component 1.

### Diarrhea, calprotectin, SCFAs, systemic inflammation and death are interrelated

**FIGURE 3:**
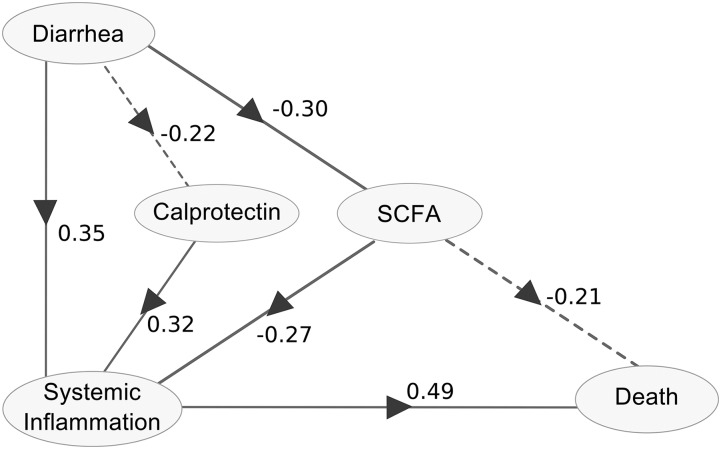
Relation between diarrhea, calprotectin, SCFAs, systemic inflammation, and death as estimated by partial least squares path modeling. Children with diarrhea status and both blood and fecal samples (*n* = 62) were included in this analysis. The path coefficients above each interconnecting arrow indicate the strength and direction of the relation between the nodes of the model. Diarrhea and calprotectin were not directly associated with death but may be linked to mortality through systemic inflammation. Similarly, SCFA shows an indirect association but may also partially contribute to death directly. Pathogens were not included in this model as this did not improve the overall fit (goodness of fit = 0.31), were not found to be associated with any nodes, and caused instability on cross-validation. Solid lines indicate a direct relation with *P* < 0.05; dashed lines indicate trends with *P* < 0.1. SCFA, short-chain fatty acid.

PLS path modeling was used to indicate the strength and direction of the relation between 5 nodes: *1*) diarrhea, *2*) calprotectin, *3*) SCFAs, *4*) markers of systemic inflammation (i.e., the 9 serum cytokines that most robustly associated with either death or diarrhea; 5 were common to both, 2 were unique to death, and 2 were unique to diarrhea), and *5*) death (**Tables 4** and **5** and **Figure 3**). Table 4 indicates the cross-correlation estimates between each variable and their attributed nodes as well as with all other model nodes. The SCFA node positively correlates with both butyrate and propionate, which indicates that patients with a high SCFA index have high values for these markers. Patients with high index values of systemic inflammation have high cytokine concentrations in their serum. Table 5 indicates the strength and directions of the calculated relation between model nodes, and these are graphically represented in Figure 3. Patients with diarrhea tended to have lower fecal calprotectin concentrations and lower SCFA concentrations but higher systemic inflammation. Patients with higher concentrations of calprotectin had a higher index of systemic inflammation, whereas higher SCFA concentrations were associated with reduced systemic inflammation. High concentrations of SCFA tended to be directly associated with less mortality, but SCFAs also showed an association with reduced systemic inflammation. This indirect relation may explain the link between SCFAs and mortality. Also, our model did not directly associate diarrhea or calprotectin with death but suggests that these markers may be linked to mortality indirectly through their association with systemic inflammation. Pathogens were not included in the final model because they did not improve the overall fit, were not found to be associated with any nodes, and caused instability on cross-validation.

**TABLE 4:** Cross-correlation between each variable and 5 main nodes of the PLS path modeling analysis^1^

	Diarrhea	Calprotectin	SCFA	Systemic inflammation	Death
Diarrhea	1	−0.22	−0.30	0.35	0.34
Calprotectin	−0.22	1	0.03	0.24	0.17
SCFA					
Propionate	−0.28	0.04	0.97	−0.32	−0.35
Butyrate	−0.30	0.02	0.98	−0.38	−0.40
Systemic inflammation					
GCSF	0.25	0.24	−0.25	0.71	0.33
IL1RA	0.11	0.25	−0.42	0.76	0.45
IL6	0.23	0.17	−0.20	0.64	0.33
IL2	0.19	0.09	−0.24	0.72	0.45
TNF-α	0.30	0.13	−0.12	0.38	0.29
TNF-β	0.23	0.13	−0.16	0.61	0.32
IL13	0.02	0.05	−0.20	0.45	0.37
IFNα2	0.43	−0.04	−0.16	0.59	0.33
IL12p70	0.22	0.22	−0.20	0.63	0.25
Death	0.34	0.17	−0.39	0.56	1

1 Cross-correlation estimates between diarrhea, calprotectin, SCFAs, markers of systemic inflammation, and death. SCFA is a composite variable of both propionate and butyrate; systemic inflammation is composed of the most robust cytokines associated with either death or diarrhea as obtained through feature selection (*n* = 9). Cross-correlation values are between 0 and 1 and indicate the correlation between each variable and model nodes (i.e. diarrhea, calprotectin, SCFAs, systemic inflammation, and death). GCSF, granulocyte-colony stimulating factor; IFN, interferon; PLS, partial least squares; SCFA, short-chain fatty acid.

**TABLE 5:** Relation between diarrhea, calprotectin, SCFA, systemic inflammation, and death as obtained from PLS path modeling^1^

				Cross-validation		
Relation between nodes	Direct	Indirect	Total	Bootstrap mean	SE	*P*^2^
Diarrhea → calprotectin	−0.222	0.000	−0.222	−0.161	0.207	0.08
Diarrhea → SCFAs	−0.298	0.000	−0.298	−0.254	0.117	0.02
Diarrhea → systemic inflammation	0.345	0.008	0.354	0.381	0.119	0.005
Calprotectin → systemic inflammation	0.324	0.000	0.324	0.308	0.116	0.006
SCFAs → systemic inflammation	−0.268	0.000	−0.268	−0.278	0.092	0.02
SCFAs → death	−0.213	−0.130	−0.343	−0.351	0.106	0.06
Systemic inflammation → death	0.485	0.000	0.485	0.497	0.099	<0.001

1 Relation estimates between diarrhea, calprotectin, the composite measures of SCFAs, and markers of systemic inflammation in relation to death. Direct and indirect relations are calculated between nodes, and total effects are the sum of these effects. SEs and bootstrap means, i.e., the mean value of the calculated total relation estimates obtained from each round of bootstrapping, were obtained through cross-validation. *P* indicates the significance of path coefficients between model nodes, which are graphically represented with arrows in Figure 3. PLS, partial least squares; SCFA, short-chain fatty acid.

2 *P* < 0.05 was considered statistically significant.

## DISCUSSION

Our study provides novel insight into the mechanisms underlying SAM-related deaths. Mortality is associated with diarrhea, low concentrations of SCFAs, and heightened intestinal and systemic inflammation.

Several studies have reported increased mortality rates in children who have both SAM and diarrhea (2, 3). Our study supports these findings, although our cohort had lower rates of diarrhea (23%) than reported frequencies of 49% (3) and 67% (2). This lower prevalence of diarrhea at admission may be due to maternal recall bias or true differences. The increased diarrhea after treatment initiation could reflect diet-induced osmotic changes.

More than 44% of children in our study harbored multiple intestinal pathogens, which may indicate colonization or active infection. Ferdous et al. (27) reported the prevalence of rotavirus at 30% and of *Shigella* at 18% in 316 rural Bangladeshi children with moderate-to-severe malnutrition and diarrhea. Amadi et al. (28) studied 194 children aged 6–24 mo n Zambia and reported the prevalence of *Cryptosporidium* at 24%, and *Salmonella* at 18%, *Giardia* at 6%, with a low prevalence of *Shigella* at 2%. We found a predominance of *Shigella*, *Giardia,* and *Campylobacter* but a low detection of enterotoxigenic *E. coli*, *Salmonella*, and *Cryptosporidium*, and a very low prevalence of norovirus and adenovirus.

Collectively, these results indicate significant variability in pathogen prevalence among children with SAM that may relate to regional differences, patient selection, sampling protocols, and analyses methods. Our study did not find associations between the presence of pathogens and diarrhea as described by Opintan et al. (7). However, our sample size was limited, and we were unable to analyze patterns of co-occurrence between pathogens. Semiquantitative analyses of stool pathogens could be useful in future studies to better differentiate between pathogens that have colonized the intestine and those actively causing overt disease in children with SAM.

Fecal calprotectin was elevated in most of our patients with ranges up to 5270 mg/kg feces. This suggests that children with SAM have high degrees of intestinal inflammation, which is consistent with a previous report (29). Interestingly, Hestvik et al. (30) described heightened concentrations of fecal calprotectin in healthy Ugandan infants with a median of 249 mg/kg feces, yet they did not find differences linked to pathogens in stool. We also did not find a clear relation between specific intestinal pathogens and fecal calprotectin; however, children with multiple pathogens did show higher concentrations. A higher calprotectin concentration was also found in children who died than in those who recovered. However, our PLS path model suggests that this link to mortality may be indirect through an association with systemic inflammation. Diarrhea tended to be associated with lower fecal calprotectin concentrations and the SCFAs butyrate and propionate, but this relation was not conclusive. With increased frequency of bowel movements, fecal markers may appear decreased because of frequent clearing of intestinal content; this may explain the high variability of fecal calprotectin and the lack of association with diarrhea or specific pathogens.

Interestingly, low concentrations of fecal butyrate and propionate were also associated with death. These byproducts of bacterial fermentation provide energy to enterocytes and modulate metabolism (12). Furthermore, SCFAs are important regulators of intestinal immunity with anti-inflammatory properties (14, 31). Also, butyrate is known to induce the cathelicidin LL-37 (32), which are antimicrobial peptides that have broad-spectrum activity against bacteria, viruses, and fungi. Patients with other intestinal diseases, such as IBD, also have reduced fecal SCFA concentrations, and this has been correlated to changes in the enteric microbiome (33). SAM patients also show significant microbiome changes that may affect SCFA production (34). Apart from microbiome-related changes, ongoing anorexia in children with SAM could have deprived the colonic microbiome of fermentable nutrients leading to lower fecal SCFA concentrations. However, the length of reported anorexia before admission did not differ between children that died or recovered and also did not correlate significantly with concentrations of SCFAs. Alternatively, starved colonic cells may uptake all available SCFAs to meet energy requirements. However, high intestinal inflammation has been associated with decreased SCFA uptake (35).

Replenishing SCFAs directly with, for example, phenyl butyrate (32, 36) or indirectly by bacterial supplementation could help reduce inflammation, increase antimicrobial peptides, and restore normal intestinal barrier function and homeostasis. To date, children with SAM have not clearly benefitted from probiotics (37), but methods for delivery and maintenance of beneficial microbiome communities may not have been fully explored.

Markers of systemic inflammation were higher in patients with SAM and associated with death and diarrhea. Malnutrition is a common cause of secondary immune deficiency, and previous studies characterizing cytokine changes showed reductions in IL2 (17, 18, 38) and IFNγ (18, 19) with inconsistent reductions in IL1 (38–40) and increases in IL10 (15, 17, 38) and TNF-α (17, 39). Compared with reference ranges (33, 41–44), >50% of patients in our cohort had higher cytokine concentrations for GCSF, IL10, IL12p40, macrophage inflammatory protein 1α, and TNF-α. The mechanisms underlying these cytokine shifts are unclear and may be related to infections, ongoing response to cell damage induced by lack of nutrients, and/or the loss of intestinal barrier function, which allows antigens to seep into the bloodstream (45). Younger age is associated with increased mortality, and younger children are known to mount differential immune responses to pathogens compared with older children (46). However, age-correction largely unchanged the cytokine patterns associated with death. Finally, SAM-associated systemic inflammation may parallel the proinflammatory shifts in cytokines that are seen in IBD with higher TNF-α, IL1, IL6, IL12, and IFNγ with decreased IL10 and TGF-β (47) concentrations. These similarities should be interpreted with caution but do warrant further investigation. Several authors have paralleled the symptoms of SAM and IBD, and this has led to the experimental use of established IBD treatments such as the anti-inflammatory agent mesalazine in patients with SAM (29, 48).

Our study was limited by a relatively small sample size and was not designed to fully investigate interactions or other cofactors of mortality such as HIV, phenotype of SAM, dehydration, electrolyte and metabolic disturbances, or inadequate antibiotic absorption. Having an age-matched control group would have been valuable, but recruitment proved infeasible because caregivers of healthy children were decidedly opposed to venipuncture. For PLS path modeling, preselecting the most robust cytokines related to death or diarrhea was done to stabilize the cross validation; this may not be a necessary with larger data sets. Better understanding of the relation between malnutrition, systemic inflammation, local inflammatory and functional changes in the intestine, and their associations with diarrhea and mortality may lead to more targeted treatments and a reduction of child mortality worldwide.

With quality research aimed at elucidating the pathophysiology of intestinal changes in SAM, new targets for interventions will be uncovered. More multicenter controlled clinical trials as well as basic mechanistic and translational research are urgently needed to provide a physiologic basis for the protocols currently used to treat SAM. Further investigating the consequences of sustained local and systemic inflammation and the links between SCFA and mortality may lead to improved clinical risk assessment and novel therapies targeting intestinal and nutritional rehabilitation in these vulnerable children.

## Footnotes

12 Abbreviations used: GC-MS, gas chromatography–mass spectrometry; GCSF, granulocyte-colony stimulating factor; IBD, inflammatory bowel disease; IFNγ, interferon γ PLS, partial least squares; SAM, severe acute malnutrition; SCFA, short-chain fatty acid.

## References

[1] Black RE, Victora CG, Walker SP, Bhutta ZA, Christian P, de Onis M, Ezzati M, Grantham-McGregor S, Katz J, Martorell R, Maternal and child undernutrition and overweight in low-income and middle-income countries. Lancet 2013;382:427–51. 23746772 10.1016/S0140-6736(13)60937-X

[2] Irena AH, Mwambazi M, Mulenga V Diarrhea is a major killer of children with severe acute malnutrition admitted to inpatient set-up in Lusaka, Zambia. Nutr J 2011;10:110. 21989455 10.1186/1475-2891-10-110 PMC3214843

[3] Talbert A, Thuo N, Karisa J, Chesaro C, Ohuma E, Ignas J, Berkley JA, Toromo C, Atkinson S, Maitland K Diarrhoea complicating severe acute malnutrition in Kenyan children: a prospective descriptive study of risk factors and outcome. PLoS One 2012;7:e38321. 22675542 10.1371/journal.pone.0038321 PMC3366921

[4] Maitland K, Berkley JA, Shebbe M, Peshu N, English M, Newton CRJC Children with severe malnutrition: can those at highest risk of death be identified with the WHO protocol? PLoS Med 2006;3:e500. 17194194 10.1371/journal.pmed.0030500 PMC1716191

[5] Heikens GT, Bunn J, Amadi B, Manary M, Chhagan M, Berkley JA, Rollins N, Kelly P, Adamczick C, Maitland K, Case management of HIV-infected severely malnourished children: challenges in the area of highest prevalence. Lancet 2008;371:1305–7. 18406865 10.1016/S0140-6736(08)60565-6

[6] World Health Organization. WHO Guideline: Updates on the management of severe acute malnutrition in infants and children. Geneva (Switzerland): World Health Organization; 2013. 24649519

[7] Opintan JA, Newman MJ, Ayeh-Kumi PF, Affrim R, Gepi-Attee R, Sevilleja JEAD, Roche JK, Nataro JP, Warren CA, Guerrant RL Pediatric diarrhea in southern Ghana: etiology and association with intestinal inflammation and malnutrition. Am J Trop Med Hyg 2010;83:936–43. 20889896 10.4269/ajtmh.2010.09-0792 PMC2946773

[8] Campbell DI, Murch SH, Elia M, Sullivan PB, Sanyang MS, Jobarteh B, Lunn PG Chronic T cell-mediated enteropathy in rural west African children: relationship with nutritional status and small bowel function. Pediatr Res 2003;54:306–11. 12788978 10.1203/01.PDR.0000076666.16021.5E

[9] Sullivan PB Studies of the small intestine in persistent diarrhea and malnutrition: the Gambian experience. J Pediatr Gastroenterol Nutr 2002;34(Suppl 1):S11–3. 12082380 10.1097/00005176-200205001-00003

[10] Fakhoury M, Negrulj R, Mooranian A, Al-Salami H Inflammatory bowel disease: clinical aspects and treatments. J Inflamm Res 2014;7:113–20. 25075198 10.2147/JIR.S65979 PMC4106026

[11] Vinolo MAR, Rodrigues HG, Nachbar RT, Curi R Regulation of inflammation by short chain fatty acids. Nutrients 2011;3:858–76. 22254083 10.3390/nu3100858 PMC3257741

[12] den Besten G, van Eunen K, Groen AK, Venema K, Reijngoud D-J, Bakker BM The role of short-chain fatty acids in the interplay between diet, gut microbiota, and host energy metabolism. J Lipid Res 2013;54:2325–40. 23821742 10.1194/jlr.R036012 PMC3735932

[13] Macia L, Tan J, Vieira AT, Leach K, Stanley D, Luong S, Maruya M, Ian McKenzie C, Hijikata A, Wong C, Metabolite-sensing receptors GPR43 and GPR109A facilitate dietary fibre-induced gut homeostasis through regulation of the inflammasome. Nat Commun 2015;6:6734. 25828455 10.1038/ncomms7734

[14] Arpaia N, Campbell C, Fan X, Dikiy S, van der Veeken J, deRoos P, Liu H, Cross JR, Pfeffer K, Coffer PJ, Metabolites produced by commensal bacteria promote peripheral regulatory T-cell generation. Nature 2013;504:451–5. 24226773 10.1038/nature12726 PMC3869884

[15] Rytter MJH, Kolte L, Briend AA, Friis H, Christensen VB The immune system in children with malnutrition:–a systematic review. PLoS One 2014;9:e105017. 25153531 10.1371/journal.pone.0105017 PMC4143239

[16] Jones KDJ, Berkley JA Severe acute malnutrition and infection. Paediatr Int Child Health 2014;34(Suppl 1):S1–29. 25475887 10.1179/2046904714Z.000000000218 PMC4266374

[17] González-Torres C, González-Martínez H, Miliar A, Nájera O, Graniel J, Firo VV, Alvarez C, Bonilla E, Rodríguez L Effect of malnutrition on the expression of cytokines involved in Th1 cell differentiation. Nutrients 2013;5:579–93. 23429441 10.3390/nu5020579 PMC3635214

[18] González-Martínez H, Rodríguez L, Nájera O, Cruz D, Miliar A, Domínguez A, Sánchez F, Graniel J, González-Torres MC Expression of cytokine mRNA in lymphocytes of malnourished children. J Clin Immunol 2008;28:593–9. 18496743 10.1007/s10875-008-9204-5

[19] Rodríguez L, González C, Flores L, Jiménez-Zamudio L, Graniel J, Ortiz R Assessment by flow cytometry of cytokine production in malnourished children. Clin Diagn Lab Immunol 2005;12:502–7. 15817757 10.1128/CDLI.12.4.502-507.2005 PMC1074380

[20] World Health Organization. Pocket book of hospital care for children: second edition [Internet]. [cited 2015 Nov 12]. Available from: http://www.who.int/maternal_child_adolescent/documents/child_hospital_care/en/.

[21] The R Foundation. The R Project for statistical computing [Internet]. [cited 2015 Sep 30]. Available from: http://www.r-project.org/.

[22] Sanchez G plsdepot: partial least squares (PLS) data analysis methods [Internet]. [cited 2015 Sep 30]. Available from: https://cran.r-project.org/web/packages/plsdepot/.

[23] Gonzalez I, Dejean S, Le Cao KA, Rohart F, Gautier B, Monget P, Coquery J, Yao FZ, Liquetey B, Benoit BL mixOmics: Omics data integration project [Internet]. [cited 2015 Oct 2]. Available from: https://cran.r-project.org/web/packages/mixOmics/.

[24] Sanchez G, Trinchera L, Russolillo G Plspm: tools for partial least squares path modeling (PLS-PM) [Internet]. [cited 2015 Sep 30]. Available from: https://cran.r-project.org/web/packages/plspm/index.html.

[25] Kuhn M, Wing J, Weston S, Williams A, Keefer C, Engelhardt ATC, Mayer Z, Kenkel B, Benesty M, Lescarbeau AZR, caret: classification and regression training [Internet]. [cited 2015 Oct 2]. Available from: https://cran.r-project.org/web/packages/caret/.

[26] Oord T, Hornung N Fecal calprotectin in healthy children. Scand J Clin Lab Invest 2014;74:254–8. 24568692 10.3109/00365513.2013.879732

[27] Ferdous F, Das SK, Ahmed S, Farzana FD, Latham JR, Chisti MJ, Ud-Din AIMS, Azmi IJ, Talukder KA, Faruque ASG Severity of diarrhea and malnutrition among under five-year-old children in rural Bangladesh. Am J Trop Med Hyg 2013;89:223–8. 23817334 10.4269/ajtmh.12-0743 PMC3741240

[28] Amadi B, Kelly P, Mwiya M, Mulwazi E, Sianongo S, Changwe F, Thomson M, Hachungula J, Watuka A, Walker-Smith J, Intestinal and systemic infection, HIV, and mortality in Zambian children with persistent diarrhea and malnutrition. J Pediatr Gastroenterol Nutr 2001;32:550–4. 11429515 10.1097/00005176-200105000-00011

[29] Jones KDJ, Hünten-Kirsch B, Laving AMR, Munyi CW, Ngari M, Mikusa J, Mulongo MM, Odera D, Nassir HS, Timbwa M, Mesalazine in the initial management of severely acutely malnourished children with environmental enteric dysfunction: a pilot randomized controlled trial. BMC Med 2014;12:133. 25189855 10.1186/s12916-014-0133-2 PMC4243388

[30] Hestvik E, Tumwine JK, Tylleskar T, Grahnquist L, Ndeezi G, Kaddu-Mulindwa DH, Aksnes L, Olafsdottir E Faecal calprotectin concentrations in apparently healthy children aged 0-12 years in urban Kampala, Uganda: a community-based survey. BMC Pediatr 2011;11:9. 21284894 10.1186/1471-2431-11-9 PMC3039585

[31] Chang PV, Hao L, Offermanns S, Medzhitov R The microbial metabolite butyrate regulates intestinal macrophage function via histone deacetylase inhibition. Proc Natl Acad Sci USA 2014;111:2247–52. 24390544 10.1073/pnas.1322269111 PMC3926023

[32] van der Does AM, Bergman P, Agerberth B, Lindbom L Induction of the human cathelicidin LL-37 as a novel treatment against bacterial infections. J Leukoc Biol 2012;92:735–42. 22701042 10.1189/jlb.0412178

[33] Bjerrum JT, Wang Y, Hao F, Coskun M, Ludwig C, Günther U, Nielsen OH Metabonomics of human fecal extracts characterize ulcerative colitis, Crohn’s disease and healthy individuals. Metabolomics 2015;11:122–33. 25598765 10.1007/s11306-014-0677-3 PMC4289537

[34] Subramanian S, Huq S, Yatsunenko T, Haque R, Mahfuz M, Alam MA, Benezra A, DeStefano J, Meier MF, Muegge BD, Persistent gut microbiota immaturity in malnourished Bangladeshi children. Nature 2014;510:417–21. 24896187 10.1038/nature13421 PMC4189846

[35] Manokas T, Fromkes JJ, Sundaram U Effect of chronic inflammation on ileal short-chain fatty acid/bicarbonate exchange. Am J Physiol Gastrointest Liver Physiol 2000;278:G585–90. 10762613 10.1152/ajpgi.2000.278.4.G585

[36] Gudmundsson GH, Bergman P, Andersson J, Raqib R, Agerberth B Battle and balance at mucosal surfaces–the story of Shigella and antimicrobial peptides. Biochem Biophys Res Commun 2010;396:116–9. 20494122 10.1016/j.bbrc.2010.03.081

[37] Kerac M, Bunn J, Seal A, Thindwa M, Tomkins A, Sadler K, Bahwere P, Collins S Probiotics and prebiotics for severe acute malnutrition (PRONUT study): a double-blind efficacy randomised controlled trial in Malawi. Lancet 2009;374:136–44. 19595348 10.1016/S0140-6736(09)60884-9

[38] Lotfy OA, Saleh WA, el-Barbari M A study of some changes of cell-mediated immunity in protein energy malnutrition. J Egypt Soc Parasitol 1998;28:413–28. 9707671

[39] Muñoz C, Arévalo M, López M, Schlesinger L Impaired interleukin-1 and tumor necrosis factor production in protein-calorie malnutrition. Nutr Res 1994;14:347–52.

[40] Bhaskaram P, Sivakumar B Interleukin-1 in malnutrition. Arch Dis Child 1986;61:182–5. 3082298 10.1136/adc.61.2.182 PMC1777567

[41] Pranzatelli MR, Tate ED, McGee NR, Colliver JA Pediatric reference ranges for proinflammatory and anti-inflammatory cytokines in cerebrospinal fluid and serum by multiplexed immunoassay. J Interferon Cytokine Res 2013;33:523–8. 23659672 10.1089/jir.2012.0132 PMC3760063

[42] Kim HO, Kim H-S, Youn J-C, Shin E-C, Park S Serum cytokine profiles in healthy young and elderly population assessed using multiplexed bead-based immunoassays. J Transl Med 2011;9:113. 21774806 10.1186/1479-5876-9-113 PMC3146842

[43] Sack U, Burkhardt U, Borte M, Schädlich H, Berg K, Emmrich F Age-dependent levels of select immunological mediators in sera of healthy children. Clin Diagn Lab Immunol 1998;5:28–32. 9455875 10.1128/cdli.5.1.28-32.1998 PMC121386

[44] Kleiner G, Marcuzzi A, Zanin V, Monasta L, Zauli G Cytokine levels in the serum of healthy subjects. Mediators Inflamm 2013;2013:434010. 23533306 10.1155/2013/434010 PMC3606775

[45] Salvatore S, Hauser B, Devreker T, Arrigo S, Vandenplas Y Chronic enteropathy and feeding in children: an update. Nutrition 2008;24:1205–16. 18621505 10.1016/j.nut.2008.04.011

[46] Philbin VJ, Levy O Developmental biology of the innate immune response: implications for neonatal and infant vaccine development. Pediatr Res 2009;65:98R–105R. 10.1203/PDR.0b013e31819f195d PMC2795575 19918215

[47] Műzes G, Molnár B, Tulassay Z, Sipos F Changes of the cytokine profile in inflammatory bowel diseases. World J Gastroenterol 2012;18:5848–61. 23139600 10.3748/wjg.v18.i41.5848 PMC3491591

[48] Hartman C, Eliakim R, Shamir R Nutritional status and nutritional therapy in inflammatory bowel diseases. World J Gastroenterol 2009;15:2570–8. 19496185 10.3748/wjg.15.2570 PMC2691486

